# Supplementary material for: Healing Pattern Analysis for Dental Implants Using the Mechano-Regulatory Tissue Differentiation Model
Source: Int J Mol Sci. 2020 Dec 2;21(23):9205. doi: 10.3390/ijms21239205 (PMC7730039; doi:10.3390/ijms21239205)
Supplement: Supplementary file 1 [file ijms-21-09205-s001.pdf]

Healing pattern analysis for dental implants using the mechano-regulatory tissue differentiation model: Supplementary

Supplementary #1:

The model in the current work was validated by the model done by Chou et al. [1], and all the settings were following their work. Figure S1 (a)(b) showed the bone healing results on the 28th day of Chou's model and our model, which is the equilibrium state and the end of tissue differentiation. It is worth noting that, the tissue phenotypes in both models were labeled in different colors. The features in both models were in good agreement. It is observed that most of the callus region was filled with immature bones; cartilage attached on the surface of the implant; also, the lower Young's modulus tissue phenotype distributed around the implant bottom, leading to a relatively lower strength.

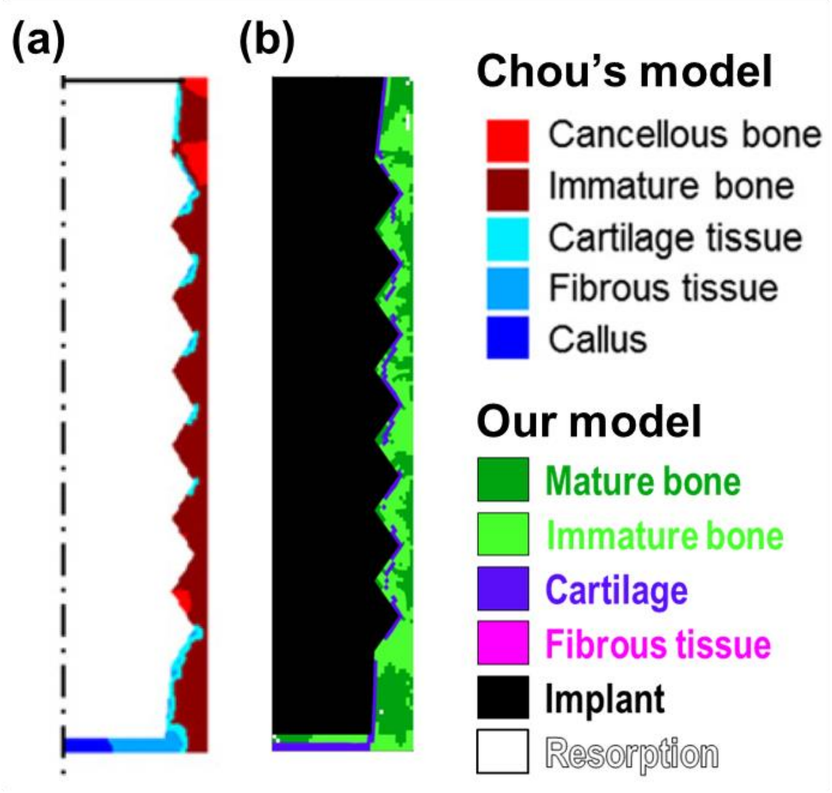

Figure S1 Bone healing results in 28th day. (a) Chou's result [1], (b) our result.

Reference

1. Chou, H.Y.; Müftü, S.Simulation of peri-implant bone healing due to immediate loading in dental implant treatments. *J. Biomech.* **2013**, *46*, 871–878, doi:10.1016/j.jbiomech.2012.12.023.
